# 10-(2-Hy­droxy­eth­yl)-9-(2-hy­droxy­phen­yl)-3,3,6,6-tetra­methyl-1,2,3,4,5,6,7,8,9,10-deca­hydro­acridine-1,8-dione

**DOI:** 10.1107/S1600536811006969

**Published:** 2011-02-26

**Authors:** Antar A. Abdelhamid, Shaaban K. Mohamed, Ali N. Khalilov, Atash V. Gurbanov, Seik Weng Ng

**Affiliations:** a Department of Organic Chemistry, Baku State University, Baku, Azerbaijan; b School of Biology, Chemistry and Material Science, Manchester Metropolitan University, Manchester, England; c Department of Chemistry, University of Malaya, 50603 Kuala Lumpur, Malaysia

## Abstract

The dihydro­pyridine ring in the title compound, C_25_H_31_NO_4_, adopts an envelope conformation with the methine C atom representing the flap. The cyclo­hexenone rings also adopt envelope conformations with the C atoms bearing the methyl C atoms representing the flaps. The phenolic hy­droxy group forms an intra­molecular hydrogen bond to one of the two keto O atoms. The hy­droxy group of the N-bonded alkyl chain forms an inter­molecular hydrogen bond to the other keto O atom of an adjacent mol­ecule. The latter hydrogen bond leads to the formation of a helical chain running along the *b* axis.

## Related literature

For a related structure, see: Jang *et al.* (2005 ▶).
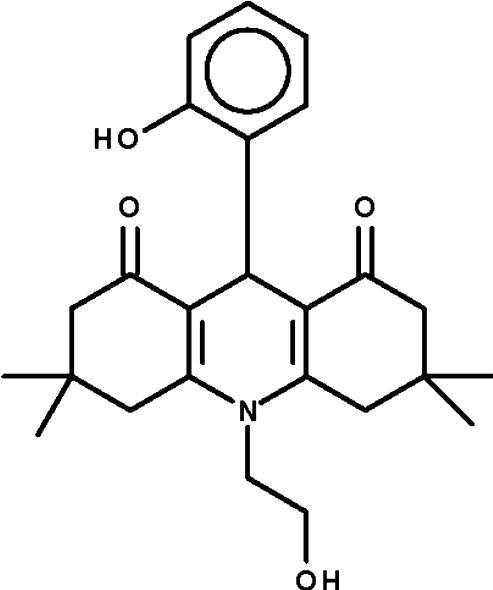


## Experimental

### 

#### Crystal data

C_25_H_31_NO_4_
*M* *_r_* = 409.51
Monoclinic, 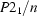

*a* = 9.7037 (2) Å
*b* = 16.5123 (3) Å
*c* = 13.8847 (3) Å
β = 102.132 (3)°
*V* = 2175.06 (8) Å^3^
*Z* = 4
Mo *K*α radiation
μ = 0.08 mm^−1^
*T* = 100 K
0.25 × 0.20 × 0.15 mm

#### Data collection

Agilent SuperNova Dual diffractometer with an Atlas detector
Absorption correction: multi-scan (*CrysAlis PRO*; Agilent, 2010 ▶) *T*_min_ = 0.979, *T*_max_ = 0.988
19082 measured reflections
4915 independent reflections
3770 reflections with *I* > 2σ(*I*)
*R*_int_ = 0.042

#### Refinement

*R*[*F* ^2^ > 2σ(*F* ^2^)] = 0.048
*wR*(*F* ^2^) = 0.123
*S* = 1.03
4915 reflections
279 parameters
2 restraints
H atoms treated by a mixture of independent and constrained refinement
Δρ_max_ = 0.27 e Å^−3^
Δρ_min_ = −0.26 e Å^−3^

### 

Data collection: *CrysAlis PRO* (Agilent, 2010 ▶); cell refinement: *CrysAlis PRO*; data reduction: *CrysAlis PRO*; program(s) used to solve structure: *SHELXS97* (Sheldrick, 2008 ▶); program(s) used to refine structure: *SHELXL97* (Sheldrick, 2008 ▶); molecular graphics: *X-SEED* (Barbour, 2001 ▶); software used to prepare material for publication: *publCIF* (Westrip, 2010 ▶).

## Supplementary Material

Crystal structure: contains datablocks global, I. DOI: 10.1107/S1600536811006969/im2270sup1.cif

Structure factors: contains datablocks I. DOI: 10.1107/S1600536811006969/im2270Isup2.hkl

Additional supplementary materials:  crystallographic information; 3D view; checkCIF report

## Figures and Tables

**Table 1:** Hydrogen-bond geometry (Å, °)

*D*—H⋯*A*	*D*—H	H⋯*A*	*D*⋯*A*	*D*—H⋯*A*
O1—H1⋯O2	0.84 (1)	1.84 (1)	2.659 (2)	166 (2)
O4—H4⋯O3^i^	0.85 (1)	1.98 (1)	2.818 (2)	166 (2)

Symmetry code: (i) 
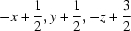
.

**Fig. 1.**
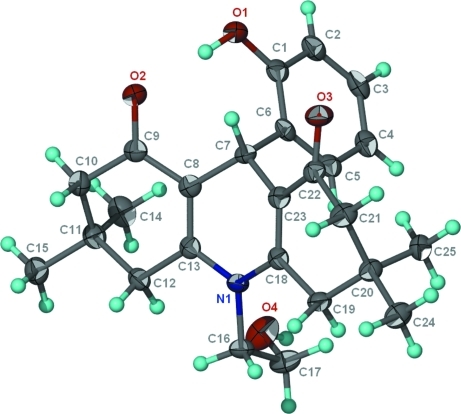
Thermal ellipsoid plot (Barbour, 2001) of C25H31NO4 at the 70% probability level; hydrogen atoms are drawn as spheres of arbitrary radius.

## supplementary crystallographic information

### Comment

Substituted benzaldehydes react with dimedone along with a primary amine to yield *N*-substituted 1,2,3,4,5,6,7,8,9,10-decahydro-acridine-1,8-diones. The title compound has a hydroxy group in 2-position of the aromatic ring. This permits intramolecular hydrogen bonding, a feature also noted in the related 9-(2,6-dihydroxyphenyl)-3,3,6,6-tetramethyl-*N*-(4-methylphenyl)-1,8-dioxo-1,2,3,4,5,6,7,8,9,10-decahydroacridine (Jang *et al.*, 2005). The second hydroxy unit in this case engages in intermolecular hydrogen bonding to afford a centrosymmetric dimer. The title compound (Scheme I, Fig. 1) has another hydroxy unit in the N bonded hydroxyethyl substituent. This groups engages in intermolecular hydrogen bond furnishing a linear chain that runs along the *c*-axis of the monoclinic unit cell.

### Experimental

Dimedone (20 mmol), salicylic aldehyde (10 mmol) and 2-amino-ethanol (10 mmol) were heated in ethanol (100 ml) for 5 h. After cooling the solution the product was collected by filtration and crystallized from ethanol; m.p. 462 K.

### Refinement

Carbon-bound H-atoms were placed in calculated positions [C—H 0.95 to 0.98 Å, *U*_iso_(H) 1.2 to 1.5*U*_eq_(C)] and were included in the refinement in the riding model approximation.

The hydroxy H-atoms were located in a difference Fourier map, and were refined with a distance restraint of O–H 0.84±0.01 Å; their temperature factors were refined.

### Crystal data

**Table:**

C_25_H_31_NO_4_	*F*(000) = 880
*M**_r_* = 409.51	*D*_x_ = 1.251 Mg m^−^^3^
Monoclinic, *P*2_1_/*n*	Mo *K*α radiation, λ = 0.71073 Å
Hall symbol: -P 2yn	Cell parameters from 6079 reflections
*a* = 9.7037 (2) Å	θ = 2.3–29.4°
*b* = 16.5123 (3) Å	µ = 0.08 mm^−^^1^
*c* = 13.8847 (3) Å	*T* = 100 K
β = 102.132 (3)°	Irregular block, light yellow
*V* = 2175.06 (8) Å^3^	0.25 × 0.20 × 0.15 mm
*Z* = 4	

### Data collection

**Table:**

Agilent SuperNova Dual diffractometer with an Atlas detector	4915 independent reflections
Radiation source: SuperNova (Mo) X-ray Source	3770 reflections with *I* > 2σ(*I*)
Mirror	*R*_int_ = 0.042
Detector resolution: 10.4041 pixels mm^-1^	θ_max_ = 27.5°, θ_min_ = 2.4°
ω scans	*h* = −12→12
Absorption correction: multi-scan (*CrysAlis PRO*; Agilent, 2010)	*k* = −21→21
*T*_min_ = 0.979, *T*_max_ = 0.988	*l* = −18→18
19082 measured reflections	

### Refinement

**Table:**

Refinement on *F*^2^	Primary atom site location: structure-invariant direct methods
Least-squares matrix: full	Secondary atom site location: difference Fourier map
*R*[*F*^2^ > 2σ(*F*^2^)] = 0.048	Hydrogen site location: inferred from neighbouring sites
*wR*(*F*^2^) = 0.123	H atoms treated by a mixture of independent and constrained refinement
*S* = 1.03	*w* = 1/[σ^2^(*F*_o_^2^) + (0.0517*P*)^2^ + 0.8053*P*] where *P* = (*F*_o_^2^ + 2*F*_c_^2^)/3
4915 reflections	(Δ/σ)_max_ = 0.001
279 parameters	Δρ_max_ = 0.27 e Å^−^^3^
2 restraints	Δρ_min_ = −0.26 e Å^−^^3^

### Fractional atomic coordinates and isotropic or equivalent isotropic displacement parameters (Å^2^)

**Table:**

	*x*	*y*	*z*	*U*_iso_*/*U*_eq_	
O1	0.48778 (12)	0.25598 (7)	0.49186 (8)	0.0245 (3)	
O2	0.71758 (12)	0.26499 (7)	0.63474 (8)	0.0239 (3)	
O3	0.32877 (12)	0.07916 (7)	0.64843 (8)	0.0252 (3)	
O4	0.36042 (14)	0.44954 (8)	0.90598 (10)	0.0357 (3)	
N1	0.45983 (13)	0.28498 (7)	0.88782 (9)	0.0174 (3)	
C1	0.37807 (16)	0.29121 (9)	0.52419 (11)	0.0198 (3)	
C2	0.28360 (17)	0.33708 (10)	0.45699 (12)	0.0242 (4)	
H2	0.2980	0.3443	0.3919	0.029*	
C3	0.16843 (17)	0.37236 (10)	0.48466 (12)	0.0245 (4)	
H3	0.1030	0.4030	0.4382	0.029*	
C4	0.14843 (17)	0.36311 (9)	0.57997 (12)	0.0232 (4)	
H4A	0.0701	0.3878	0.5993	0.028*	
C5	0.24353 (16)	0.31759 (9)	0.64673 (11)	0.0201 (3)	
H5	0.2297	0.3118	0.7121	0.024*	
C6	0.35895 (16)	0.28008 (9)	0.62057 (11)	0.0181 (3)	
C7	0.46282 (16)	0.22884 (9)	0.69374 (11)	0.0173 (3)	
H7	0.5066	0.1884	0.6557	0.021*	
C8	0.57885 (16)	0.27978 (9)	0.75365 (11)	0.0182 (3)	
C9	0.70525 (17)	0.29325 (9)	0.71557 (11)	0.0195 (3)	
C10	0.82323 (16)	0.34044 (9)	0.77743 (12)	0.0212 (3)	
H10A	0.8826	0.3637	0.7344	0.025*	
H10B	0.8828	0.3034	0.8247	0.025*	
C11	0.76993 (17)	0.40884 (9)	0.83440 (12)	0.0216 (3)	
C12	0.67029 (16)	0.37278 (9)	0.89612 (11)	0.0204 (3)	
H12A	0.7278	0.3468	0.9554	0.024*	
H12B	0.6167	0.4175	0.9186	0.024*	
C13	0.56744 (16)	0.31146 (9)	0.84253 (11)	0.0173 (3)	
C14	0.69201 (19)	0.47206 (10)	0.76251 (13)	0.0298 (4)	
H14A	0.7563	0.4945	0.7236	0.045*	
H14B	0.6581	0.5156	0.7995	0.045*	
H14C	0.6117	0.4465	0.7184	0.045*	
C15	0.89404 (18)	0.44878 (10)	0.90356 (13)	0.0273 (4)	
H15A	0.9575	0.4726	0.8649	0.041*	
H15B	0.9452	0.4081	0.9487	0.041*	
H15C	0.8592	0.4913	0.9415	0.041*	
C16	0.43798 (17)	0.32332 (9)	0.97968 (11)	0.0204 (3)	
H16A	0.5266	0.3500	1.0131	0.025*	
H16B	0.4154	0.2808	1.0243	0.025*	
C17	0.32055 (19)	0.38537 (10)	0.96141 (12)	0.0266 (4)	
H17A	0.2323	0.3602	0.9249	0.032*	
H17B	0.3039	0.4060	1.0249	0.032*	
C18	0.38348 (16)	0.21533 (9)	0.85240 (11)	0.0169 (3)	
C19	0.29342 (16)	0.17702 (9)	0.91636 (11)	0.0195 (3)	
H19A	0.2381	0.2200	0.9405	0.023*	
H19B	0.3559	0.1523	0.9745	0.023*	
C20	0.19150 (17)	0.11202 (9)	0.86392 (11)	0.0203 (3)	
C21	0.27110 (18)	0.05689 (9)	0.80557 (12)	0.0236 (4)	
H21A	0.3488	0.0292	0.8514	0.028*	
H21B	0.2063	0.0150	0.7708	0.028*	
C22	0.33022 (16)	0.10522 (9)	0.73201 (11)	0.0193 (3)	
C23	0.39203 (16)	0.18370 (9)	0.76380 (11)	0.0179 (3)	
C24	0.13741 (18)	0.06237 (10)	0.94152 (13)	0.0259 (4)	
H24A	0.0883	0.0981	0.9795	0.039*	
H24B	0.2172	0.0365	0.9859	0.039*	
H24C	0.0722	0.0207	0.9088	0.039*	
C25	0.06713 (17)	0.15154 (10)	0.79316 (12)	0.0238 (4)	
H25A	0.1023	0.1835	0.7439	0.036*	
H25B	0.0162	0.1870	0.8303	0.036*	
H25C	0.0033	0.1094	0.7600	0.036*	
H1	0.5572 (17)	0.2508 (14)	0.5393 (12)	0.051 (7)*	
H4	0.2939 (18)	0.4842 (11)	0.8949 (16)	0.053 (7)*	

### Atomic displacement parameters (Å^2^)

**Table:**

	*U*^11^	*U*^22^	*U*^33^	*U*^12^	*U*^13^	*U*^23^
O1	0.0232 (6)	0.0345 (7)	0.0163 (6)	−0.0009 (5)	0.0056 (5)	−0.0008 (5)
O2	0.0219 (6)	0.0323 (6)	0.0188 (6)	−0.0007 (5)	0.0074 (5)	−0.0029 (5)
O3	0.0310 (7)	0.0246 (6)	0.0225 (6)	−0.0054 (5)	0.0110 (5)	−0.0070 (5)
O4	0.0373 (8)	0.0282 (7)	0.0454 (8)	0.0109 (6)	0.0175 (6)	0.0102 (6)
N1	0.0183 (7)	0.0202 (6)	0.0141 (6)	0.0011 (5)	0.0040 (5)	−0.0022 (5)
C1	0.0210 (8)	0.0203 (8)	0.0181 (7)	−0.0056 (6)	0.0041 (6)	−0.0021 (6)
C2	0.0262 (9)	0.0266 (8)	0.0181 (8)	−0.0098 (7)	0.0009 (7)	0.0014 (7)
C3	0.0218 (8)	0.0229 (8)	0.0244 (8)	−0.0047 (7)	−0.0049 (7)	0.0027 (7)
C4	0.0208 (8)	0.0212 (8)	0.0261 (8)	−0.0010 (7)	0.0013 (7)	−0.0031 (7)
C5	0.0213 (8)	0.0196 (8)	0.0190 (8)	−0.0039 (6)	0.0034 (6)	−0.0027 (6)
C6	0.0191 (8)	0.0170 (7)	0.0170 (7)	−0.0059 (6)	0.0007 (6)	−0.0024 (6)
C7	0.0186 (8)	0.0193 (7)	0.0151 (7)	−0.0003 (6)	0.0057 (6)	−0.0021 (6)
C8	0.0184 (8)	0.0186 (7)	0.0174 (7)	0.0020 (6)	0.0036 (6)	0.0015 (6)
C9	0.0201 (8)	0.0203 (8)	0.0182 (8)	0.0035 (6)	0.0039 (6)	0.0024 (6)
C10	0.0187 (8)	0.0245 (8)	0.0204 (8)	−0.0006 (7)	0.0044 (6)	0.0013 (7)
C11	0.0209 (8)	0.0203 (8)	0.0230 (8)	−0.0012 (6)	0.0029 (7)	−0.0004 (7)
C12	0.0193 (8)	0.0219 (8)	0.0191 (8)	0.0024 (6)	0.0020 (6)	−0.0032 (6)
C13	0.0167 (8)	0.0174 (7)	0.0172 (7)	0.0039 (6)	0.0024 (6)	0.0012 (6)
C14	0.0313 (10)	0.0240 (8)	0.0322 (9)	0.0013 (7)	0.0021 (8)	0.0045 (7)
C15	0.0231 (9)	0.0280 (9)	0.0305 (9)	−0.0042 (7)	0.0048 (7)	−0.0041 (7)
C16	0.0243 (8)	0.0234 (8)	0.0144 (7)	0.0007 (7)	0.0058 (6)	−0.0040 (6)
C17	0.0338 (10)	0.0248 (8)	0.0244 (8)	0.0055 (7)	0.0132 (7)	−0.0010 (7)
C18	0.0154 (7)	0.0170 (7)	0.0179 (7)	0.0033 (6)	0.0026 (6)	0.0010 (6)
C19	0.0218 (8)	0.0223 (8)	0.0152 (7)	0.0017 (6)	0.0059 (6)	−0.0003 (6)
C20	0.0220 (8)	0.0204 (8)	0.0201 (8)	−0.0011 (6)	0.0080 (7)	0.0008 (6)
C21	0.0286 (9)	0.0186 (8)	0.0257 (8)	0.0004 (7)	0.0105 (7)	−0.0003 (7)
C22	0.0188 (8)	0.0201 (7)	0.0196 (7)	0.0026 (6)	0.0057 (6)	−0.0005 (6)
C23	0.0179 (8)	0.0185 (7)	0.0176 (7)	0.0025 (6)	0.0047 (6)	0.0004 (6)
C24	0.0299 (9)	0.0240 (8)	0.0268 (8)	−0.0012 (7)	0.0127 (7)	0.0031 (7)
C25	0.0208 (8)	0.0281 (8)	0.0230 (8)	−0.0031 (7)	0.0058 (7)	0.0000 (7)

### Geometric parameters (Å, °)

**Table:**

O1—C1	1.3690 (19)	C12—C13	1.504 (2)
O1—H1	0.841 (10)	C12—H12A	0.9900
O2—C9	1.2442 (18)	C12—H12B	0.9900
O3—C22	1.2349 (18)	C14—H14A	0.9800
O4—C17	1.411 (2)	C14—H14B	0.9800
O4—H4	0.852 (9)	C14—H14C	0.9800
N1—C13	1.3975 (19)	C15—H15A	0.9800
N1—C18	1.400 (2)	C15—H15B	0.9800
N1—C16	1.4785 (18)	C15—H15C	0.9800
C1—C2	1.388 (2)	C16—C17	1.514 (2)
C1—C6	1.401 (2)	C16—H16A	0.9900
C2—C3	1.384 (2)	C16—H16B	0.9900
C2—H2	0.9500	C17—H17A	0.9900
C3—C4	1.386 (2)	C17—H17B	0.9900
C3—H3	0.9500	C18—C23	1.355 (2)
C4—C5	1.384 (2)	C18—C19	1.510 (2)
C4—H4A	0.9500	C19—C20	1.535 (2)
C5—C6	1.393 (2)	C19—H19A	0.9900
C5—H5	0.9500	C19—H19B	0.9900
C6—C7	1.528 (2)	C20—C24	1.532 (2)
C7—C23	1.502 (2)	C20—C21	1.531 (2)
C7—C8	1.508 (2)	C20—C25	1.533 (2)
C7—H7	1.0000	C21—C22	1.502 (2)
C8—C13	1.366 (2)	C21—H21A	0.9900
C8—C9	1.451 (2)	C21—H21B	0.9900
C9—C10	1.497 (2)	C22—C23	1.457 (2)
C10—C11	1.530 (2)	C24—H24A	0.9800
C10—H10A	0.9900	C24—H24B	0.9800
C10—H10B	0.9900	C24—H24C	0.9800
C11—C15	1.524 (2)	C25—H25A	0.9800
C11—C14	1.530 (2)	C25—H25B	0.9800
C11—C12	1.540 (2)	C25—H25C	0.9800
			
C1—O1—H1	109.6 (16)	H14A—C14—H14C	109.5
C17—O4—H4	108.6 (15)	H14B—C14—H14C	109.5
C13—N1—C18	119.28 (12)	C11—C15—H15A	109.5
C13—N1—C16	120.69 (12)	C11—C15—H15B	109.5
C18—N1—C16	119.57 (12)	H15A—C15—H15B	109.5
O1—C1—C2	117.44 (14)	C11—C15—H15C	109.5
O1—C1—C6	121.71 (14)	H15A—C15—H15C	109.5
C2—C1—C6	120.83 (15)	H15B—C15—H15C	109.5
C3—C2—C1	120.06 (15)	N1—C16—C17	112.62 (13)
C3—C2—H2	120.0	N1—C16—H16A	109.1
C1—C2—H2	120.0	C17—C16—H16A	109.1
C2—C3—C4	120.13 (16)	N1—C16—H16B	109.1
C2—C3—H3	119.9	C17—C16—H16B	109.1
C4—C3—H3	119.9	H16A—C16—H16B	107.8
C5—C4—C3	119.46 (15)	O4—C17—C16	108.47 (13)
C5—C4—H4A	120.3	O4—C17—H17A	110.0
C3—C4—H4A	120.3	C16—C17—H17A	110.0
C4—C5—C6	121.74 (15)	O4—C17—H17B	110.0
C4—C5—H5	119.1	C16—C17—H17B	110.0
C6—C5—H5	119.1	H17A—C17—H17B	108.4
C5—C6—C1	117.76 (14)	C23—C18—N1	120.45 (13)
C5—C6—C7	121.80 (13)	C23—C18—C19	121.74 (14)
C1—C6—C7	120.43 (14)	N1—C18—C19	117.81 (12)
C23—C7—C8	108.04 (12)	C18—C19—C20	114.13 (12)
C23—C7—C6	112.22 (12)	C18—C19—H19A	108.7
C8—C7—C6	111.70 (12)	C20—C19—H19A	108.7
C23—C7—H7	108.2	C18—C19—H19B	108.7
C8—C7—H7	108.3	C20—C19—H19B	108.7
C6—C7—H7	108.2	H19A—C19—H19B	107.6
C13—C8—C9	120.37 (14)	C24—C20—C21	109.84 (13)
C13—C8—C7	121.27 (13)	C24—C20—C25	109.79 (13)
C9—C8—C7	118.35 (13)	C21—C20—C25	109.31 (13)
O2—C9—C8	121.33 (14)	C24—C20—C19	108.81 (13)
O2—C9—C10	120.38 (14)	C21—C20—C19	108.68 (13)
C8—C9—C10	118.27 (13)	C25—C20—C19	110.39 (13)
C9—C10—C11	112.33 (13)	C22—C21—C20	110.56 (13)
C9—C10—H10A	109.1	C22—C21—H21A	109.5
C11—C10—H10A	109.1	C20—C21—H21A	109.5
C9—C10—H10B	109.1	C22—C21—H21B	109.5
C11—C10—H10B	109.1	C20—C21—H21B	109.5
H10A—C10—H10B	107.9	H21A—C21—H21B	108.1
C15—C11—C14	109.44 (14)	O3—C22—C23	121.15 (14)
C15—C11—C10	109.70 (13)	O3—C22—C21	121.56 (14)
C14—C11—C10	109.92 (13)	C23—C22—C21	117.28 (13)
C15—C11—C12	108.79 (13)	C18—C23—C22	121.21 (13)
C14—C11—C12	110.17 (13)	C18—C23—C7	121.36 (14)
C10—C11—C12	108.80 (12)	C22—C23—C7	117.42 (13)
C13—C12—C11	114.45 (12)	C20—C24—H24A	109.5
C13—C12—H12A	108.6	C20—C24—H24B	109.5
C11—C12—H12A	108.6	H24A—C24—H24B	109.5
C13—C12—H12B	108.6	C20—C24—H24C	109.5
C11—C12—H12B	108.6	H24A—C24—H24C	109.5
H12A—C12—H12B	107.6	H24B—C24—H24C	109.5
C8—C13—N1	119.79 (14)	C20—C25—H25A	109.5
C8—C13—C12	122.23 (13)	C20—C25—H25B	109.5
N1—C13—C12	117.95 (13)	H25A—C25—H25B	109.5
C11—C14—H14A	109.5	C20—C25—H25C	109.5
C11—C14—H14B	109.5	H25A—C25—H25C	109.5
H14A—C14—H14B	109.5	H25B—C25—H25C	109.5
C11—C14—H14C	109.5		

### Hydrogen-bond geometry (Å, °)

**Table:**

*D*—H···*A*	*D*—H	H···*A*	*D*···*A*	*D*—H···*A*
O1—H1···O2	0.84 (1)	1.84 (1)	2.659 (2)	166 (2)
O4—H4···O3^i^	0.85 (1)	1.98 (1)	2.818 (2)	166 (2)

Symmetry codes: (i) −*x*+1/2, *y*+1/2, −*z*+3/2.
